# Supplementary figures and images for: Low carbon economic dispatch of integrated energy system based on coupled operation of OCPP-P2G-CHP
Source: PLoS One. 2025 May 19;20(5):e0322992. doi: 10.1371/journal.pone.0322992 (PMC12088528; doi:10.1371/journal.pone.0322992)

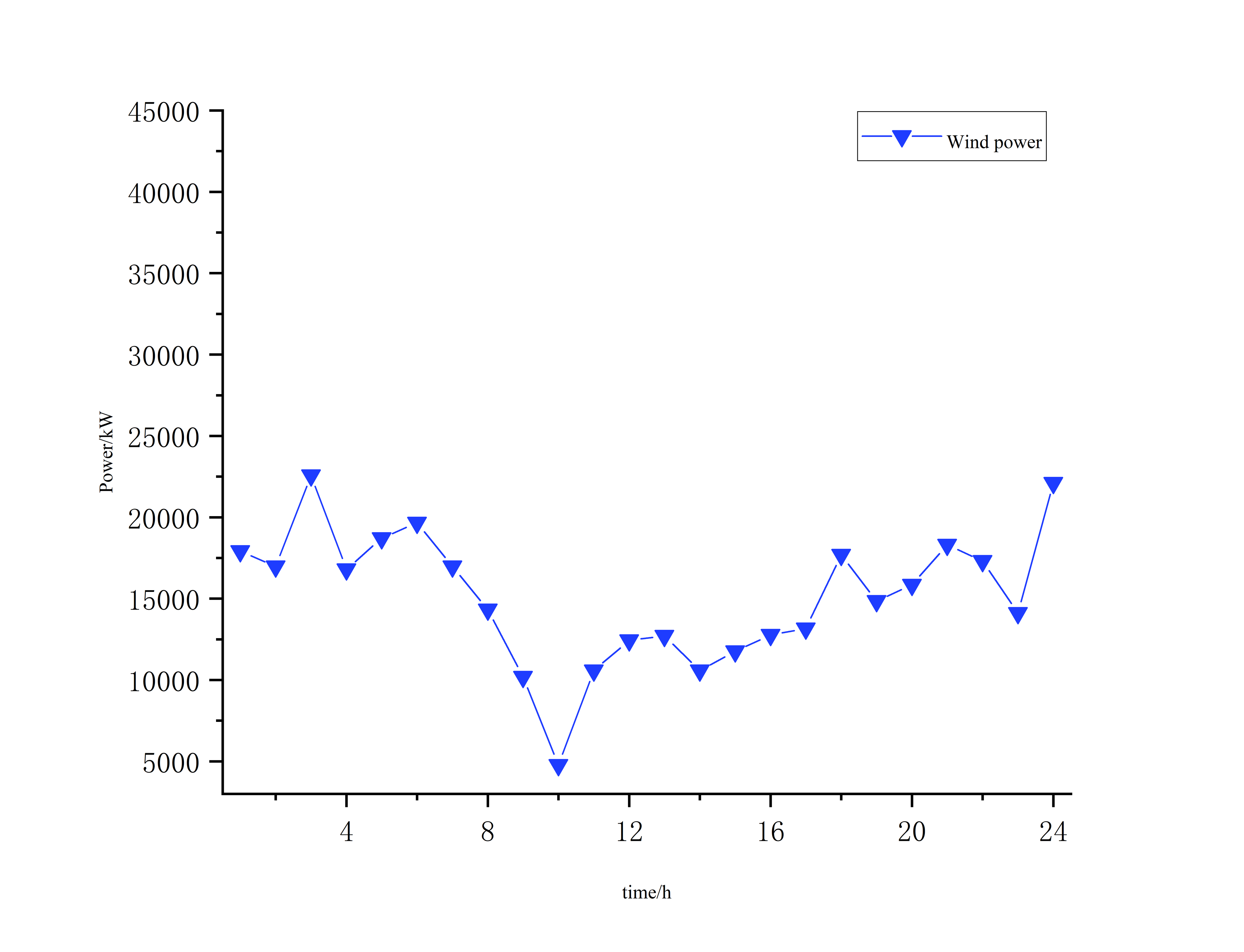

Supplement: S1 Fig — (TIF) [file pone.0322992.s001.tif]

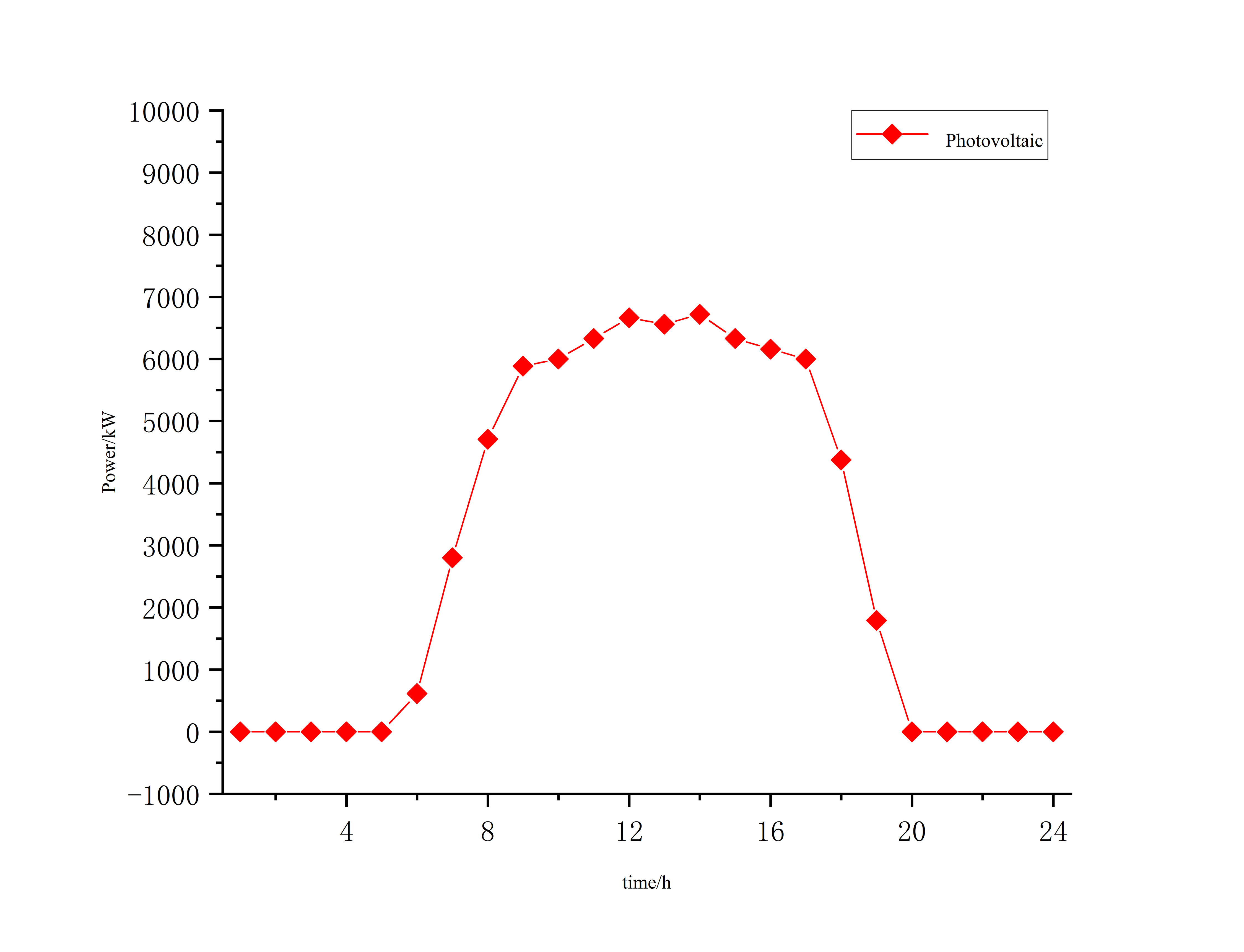

Supplement: S2 Fig — (TIF) [file pone.0322992.s002.tif]

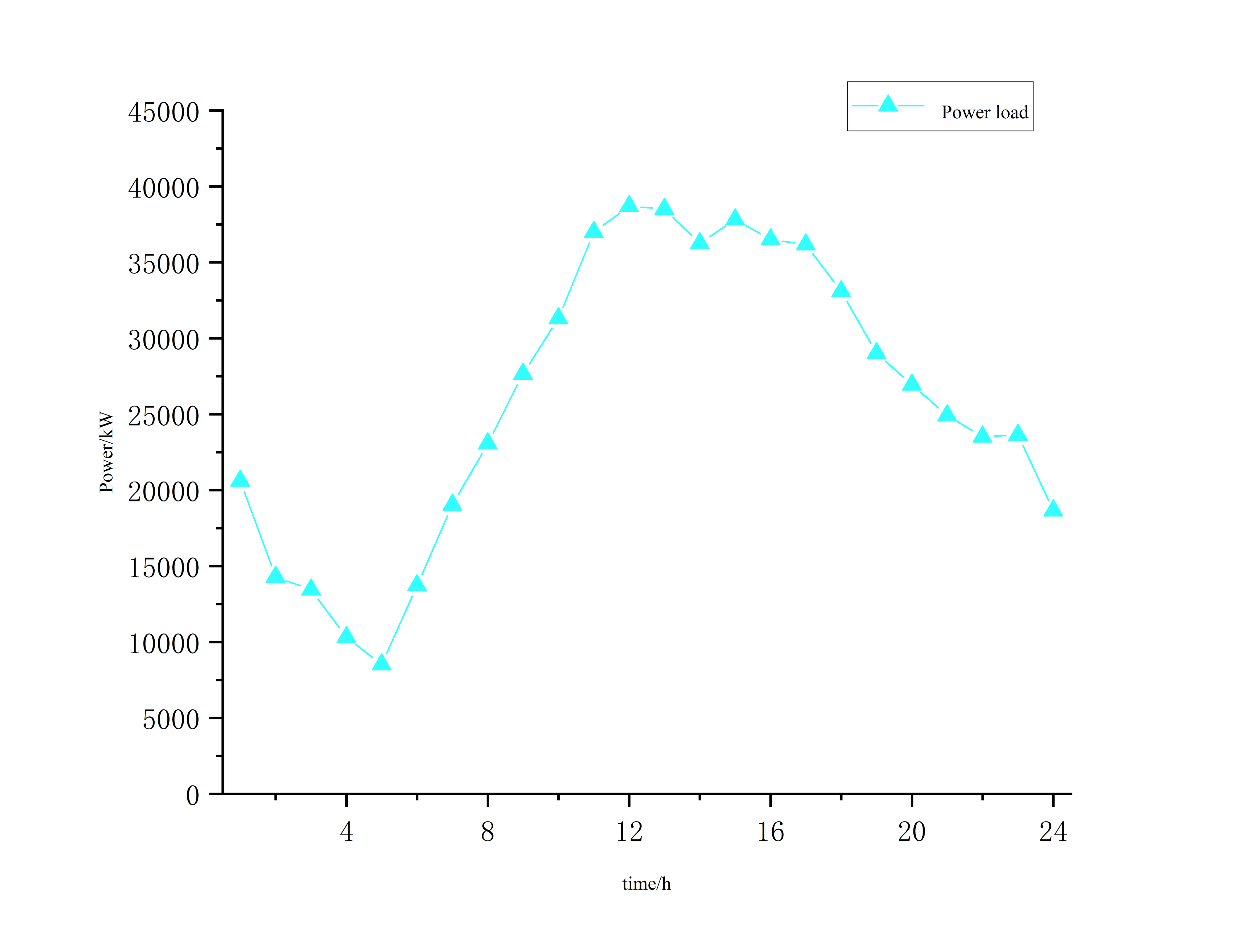

Supplement: S3 Fig — (TIF) [file pone.0322992.s003.tif]

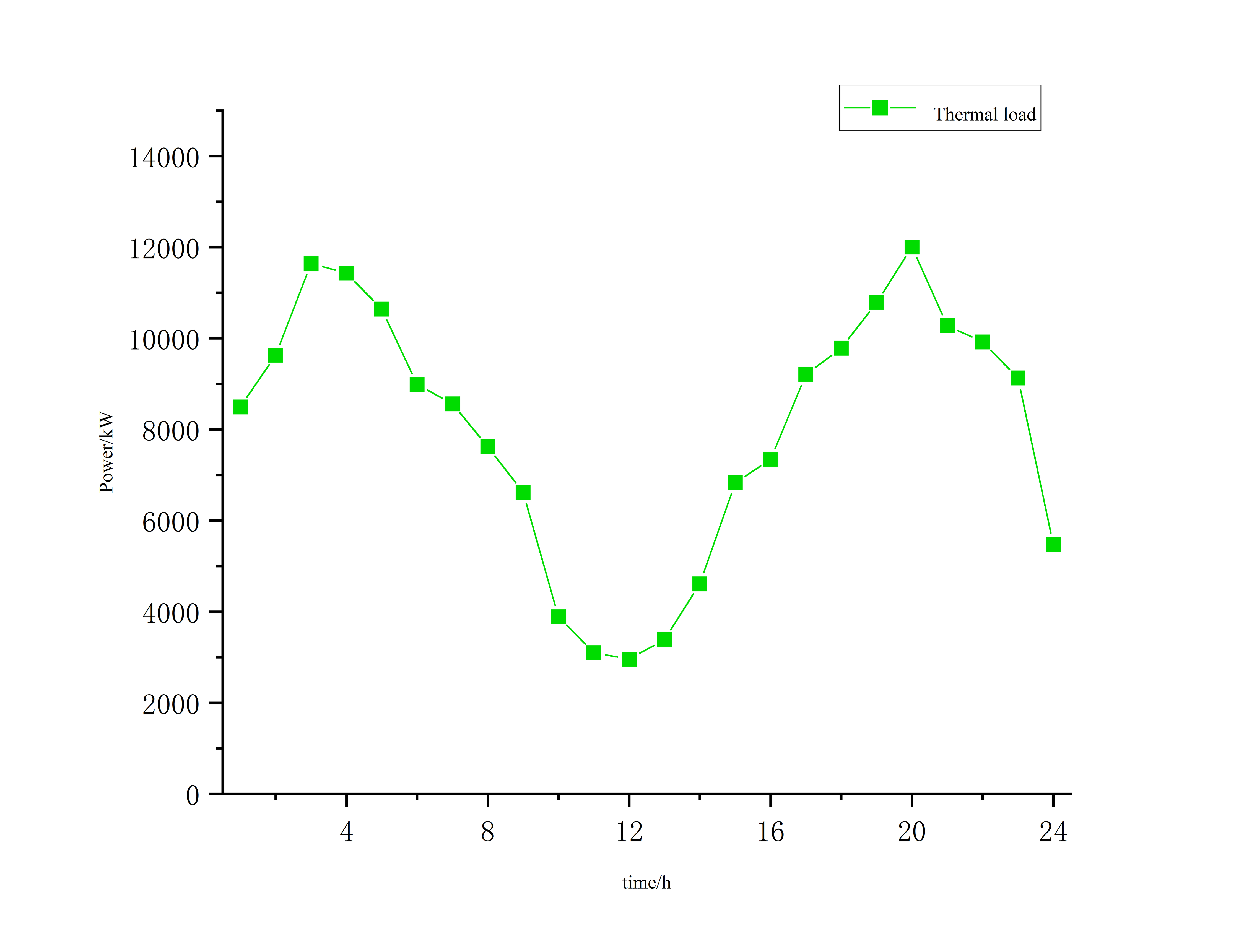

Supplement: S4 Fig — (TIF) [file pone.0322992.s004.tif]
