# Supplementary material for: Low carbon economic dispatch of integrated energy system based on coupled operation of OCPP-P2G-CHP
Source: PLoS One. 2025 May 19;20(5):e0322992. doi: 10.1371/journal.pone.0322992 (PMC12088528; doi:10.1371/journal.pone.0322992)
Supplement: Attachment — Submitted filename: Response to Reviewers.docx [file pone.0322992.s007.docx]

Dear Editors and Reviewers:

Thank you for your letter and for the reviewers’ comments concerning our manuscript entitled “Low Carbon Economic Dispatch of Integrated Energy System Based on Coupled Operation of OCPP-P2G-CHP”.Those comments are all valuable and very helpful for revising and improving our paper, as well as the important guiding significance to our researches. We have studied comments carefully and have made correction which we hope meet with approval.

Responds to the reviewer’s comments:

Reviewer #1:

*1. The various parts of the model mentioned in the paper are closely related, but all of them are modeled using fixed-value linear models, without considering the dynamic characteristics of the model.*

The author's answer: Thank you very much for this suggestion.We are very sorry for our negligence of the dynamic characteristics of the model. We have made corrections based on your comments. We modified the model for OCPP, CHP, and P2G, and added climbing constraints on the basis of the original model in order to enhance the model's portrayal of the dynamic process and reflect the dynamic regulation capability of the core technology of this paper more realistically.

*2. The following related research can be compared a) Two-Stage Coordinated Operation of A Green Multi-Energy Ship Microgrid With Underwater Radiated Noise by Distributed Stochastic Approach b) A multi-stage stochastic dispatching method for electricity-hydrogen integrated energy systems driven by model and data.*

The author's answer: We couldn't agree with you more on this suggestion. Considering your suggestion, we have compared these studies and placed them in the literature review of this paper. We refer to these papers as references 18 and 19. We were inspired by these researches, which we read carefully and revised the article reasonably. And, these studies have also provided us with directions for future research. We are very grateful to you for this suggestion.

*3. The innovation of the article is not strong enough. The oxygen-enriched combustion power plant (OCPP) is the core of the innovation of this paper, but the modeling is too rough. Please enrich the modeling of this part.*

The author's answer: Thank you very much for your professional comments on our article. And we deeply apologize for the shortcomings in the modeling of OCPP. Following your suggestions, we have supplemented and enriched the original model and added some textual narratives, which we hope will meet your requirements.

*4. The simulation part of this paper lacks the power system topology diagram and the photovoltaic and wind power data used lacks source citations.*

The author's answer: We think your suggestion is very useful. And we apologize for the lack of power system topology diagrams. Therefore, we have added the power system topology diagram in the simulation section, labeled as Fig. 6, and the energy flow corresponds to Fig. 2. Forecasts for wind, photovoltaic, and loads are from Ref. 40, with cited sources noted in the article.

*5. Scheduling is the title of this paper but the simulation part is too rough. One hour as the scheduling step is a bit too long for this model. Please reconsider the scheduling step.*

The author's answer: Thank you for your careful analysis of our simulation section, we think your suggestion makes a lot of sense! We are equally in strong agreement about the simulation portion of the problem, and we are acutely aware of the issues regarding the steps of the program. However, due to the limitations of the data and literature on wind, photovoltaic and load forecasting, we found it difficult to undertake this work because it was beyond the scope of our simulations. To address the roughness of the simulation part, we added the hydrogen power balance, labeled Figure 15, to the analysis of the scheduling results and analyzed it. The above deficiencies are also the direction of our future thesis research. We hope to have your understanding.

*6. Figures 2, 3, 5 and 6 in this paper are too rough. Please redraw them.*

The author's answer: Thank you very much for your careful examination of our figures. Taking into account the reviewers' suggestions for the figures, we have re-processed the figures for clarity. We apologize to the reviewers for any reading difficulties.

Reviewer #2:

*1. Abstract: what is the novelty addressed by your research? Please state explicitly the novelty involved in your approach. Quantitative data/conclusions are also welcome.*

The author's answer: Thank you for this suggestion.Based on your suggestions for the abstract, we have revised it to better convey the innovative contribution and practical significance of the article.

*2. Avoid repeating frequently an acronym and its definition. E.g., Integrated Energy Systems (IES).*

The author's answer: We sincerely apologize for our oversight. We appreciate your reminder, which prompted us to review the entire text and implement the necessary corrections.

*3. Recommendation to include in the paper a nomenclature of the used symbols & acronyms.*

The author's answer: Your suggestion is very rigorous and reasonable.The manuscript has been supplemented with an acronym nomenclature. However, due to the extensive number of specialized symbols utilized within the article, providing a comprehensive list would be excessively space-consuming. Consequently, we have opted not to include a detailed explanation of each symbol; instead, we have clarified the meaning of the symbols upon their initial occurrence in the text. We regret this decision and kindly request the reviewers' understanding.

*4. Proposal to consider the much significant syntagma “Author et al. [x]...” instead of anonymous “In [x]…”, “Ref [x] presents”, etc.*

The author's answer: We express our sincere appreciation to the reviewers for their valuable suggestion. Consequently, we have implemented grammatical revisions in the literature review section of the manuscript.

*5. Solve several typing mistakes, e.g. “processes. [26].”, “is the Net”, “boilers(GB)modeling”, “boilers(EB)”, etc. Check carefully the entire manuscript for other similar mistakes.*

The author's answer: Thanks for your careful checks.We feel sorry for our carelessness. We have corrected it and we also feel great thanks for your point out. We apologize for any reading difficulties you may have had!

*6. Typically, the paper structure (its sections) is briefly presented at the end of Introduction.*

The author's answer: Thank you for this suggestion, for which we give a brief description of the structure of the paper at the end of the introduction.

*7. Define all symbols used in equations and/or include their definition / significance in Nomenclature.*

The author's answer: Thank you for this suggestion, and again, for the sake of having too many symbols, we've only described them below the first appearance of the symbol. We hope you understand, and we apologize for this.

*8. Increase the quality (resolution) of all figures, they are in current state at the legibility limit.*

The author's answer: We apologize for the image clarity issue. For this reason, we have improved the clarity of all the images in the article to ensure the smoothness of your subsequent reading. We hope this error has not caused you any reading displeasure.

*9. Fig. 2: the role of the CC unit in the OCPP system in not clearly explain (it is isolated form the rest of the components, there is only a power input, without a specific output). Similarly for other figures, where the local outputs should be also indicated.*

The author's answer: Thank you very much for this suggestion for our paper. To this end we have revised Figure 2 to clearly illustrate the role of the CC unit in the OCPP system, including its inputs and outputs. For clarity, a corresponding explanation has been added to the text. Similar changes have also been made to the others.

*10. Justify the selection of curves in Fig. 5 and Fig. 6: what they represent in this specific case study? Are they coming from measurements? Please clarify it!*

The author's answer: The photovoltaic power curve in Figure 5 shows the variation of solar power generation over time, usually peaking during the day and dropping to zero at night. The wind power curve shows the variation of wind power generation over time, which is usually affected by changes in wind speed and is highly volatile. The thermal load curve shows the variation of the system's demand for thermal energy, related to industrial processes, heating or cooling needs. The electric load curve shows the variation in the system's demand for electricity, usually associated with industrial, commercial, and residential use. The natural gas load curve in Figure 6 shows the variation in system demand for natural gas, which may be associated with power generation, heating, or industrial processes. The photovoltaic, wind, and electric load data in Figure 5 can be used to optimize the synergistic operation of renewable energy sources with fossil energy sources. The natural gas load data in Figure 6 can be combined with the heat load data in Figure 5 to optimize the operation of combined heat and power (CHP) systems. Forecasts for wind, photovoltaic, and loads are from Ref. 40, with cited sources noted in the article. The data is from a comprehensive park and is indicative and representative.

*11. The literature review: an appropriate systematization of the identified solutions and challenges in the addressed topic is more valuable instead of presenting one by one the paper results.*

The author's answer: In response to this suggestion of yours, we have revised the literature review to specify the resolution and contribution of the references.

*12. A flowchart of the proposed approach is welcomed before introducing all mathematical issues.*

The author's answer: Thank you very much for this suggestion, so we have added a flowchart of the proposed approach in the introduction section.

*13. Use appropriate reference(s) whenever unproven equations are introduced (i.e., the large majority of the equations). Make a clear difference between existing and proposed equations.*

The author's answer: We couldn't agree more with you on this suggestion. Therefore we have added references [32-38] near some of the equations to distinguish the existing equations from the proposed ones.

*14. Objective function: some clarification are needed related to the cost optimization concept by considering the whole life cycle cost of the system, not only the operating cost. Or, at least state the limits of the proposed approach.*

The author's answer: Thank you very much for your professional comments on our article. As you are concerned, this issue needs to be addressed. However, due to the limitations of the available information, we are not able to optimize the whole life cycle cost of the system. Therefore, we have explained this shortcoming of the article in the conclusion section. Thank you again for this very valuable suggestion, which provides us with a direction for future research.

*15. 6. Conclusion: proposal to rename it “6. Conclusions” as several conclusions are drawn. The limits of the proposed system and approach, as well as future works, should be highlighted here.*

The author's answer: We were really sorry for our careless mistakes. Thank you for your reminder. We have added at the conclusion the shortcomings of the research methodology and the directions for future research needed.

Finally, thank you again for your suggestions regarding with our paper. I look forward to hearing from you.

Yours sincerely,

Jingjing Ma

26, February,2025

Hubei University of Technology
